# Trapped fourth ventricle: to stent, shunt, or fenestrate—a systematic review and individual patient data meta-analysis

**DOI:** 10.1007/s10143-023-01957-x

**Published:** 2023-01-28

**Authors:** Yasmin Sadigh, Colin van Surksum, Philip H. D. Schröder, Ayca Cozar, Dalila Khandour, Lailla Talbi, Jochem K. H. Spoor, Oscar H. J. Eelkman Rooda, Victor Volovici, Marie-Lise C. van Veelen

**Affiliations:** 1 Department of Pediatric Neurosurgery, Sophia Children’s Hospital, Erasmus MC, Rotterdam, The Netherlands; 2 Department of Neurosurgery, Erasmus MC Stroke Center, Erasmus MC Rotterdam, Rotterdam, The Netherlands; 3 Center for Medical Decision Making, Erasmus MC Rotterdam, Rotterdam, The Netherlands; 4 Departments of Neurosurgery, Pediatric Neurosurgery and Public Health, Erasmus MC University Medical Center, Dr Molewaterplein 40, 3015 GD Rotterdam, The Netherlands

**Keywords:** Trapped fourth ventricle, Isolated fourth ventricle, Endoscopy, Microsurgery, Shunt placement

## Abstract

Trapped or isolated fourth ventricle (TFV) is a rare but critical neurosurgical condition, mostly occurring in pediatric patients, caused by a blockage of the in- and outlets of the fourth ventricle. The purpose of this study is to review all available data on the treatment options of TFV and to compare their safety, efficacy, and durability. MEDLINE, Embase, and Google Scholar were searched from inception to September 13, 2022, for prospective or retrospective cohorts, case-control studies, case series or case reports, reporting detailing outcomes of TFV patients, treated with an endoscopic-, microsurgical-, shunt placement,- or hybrid approaches to TFV. All authors were contacted to provide individual patient data. Eighty-seven articles (314 patients) were included in the individual patient data meta-analysis (IPD) and 9 (151 patients) in the cohort meta-analysis. The IPD revealed that primary endoscopic (*aOR* 0.21; [95% *CI* 0.08–0.57]) and microsurgical interventions (*aOR* 0.21; [95% *CI* 0.05–0.82]) were associated with a significantly lower revision rate, compared to shunt placement, when adjusted for confounders. Endoscopy was also associated with a significantly higher rate of clinical improvement (*aOR* 4.56; [95% *CI* 1.2–18]). The meta-analysis revealed no significant difference in revision rate between the endoscopic (0.33 [95% *CI* 0.0–0.52]) and shunt group (0.44 [95% *CI* 0.0–1.0]). Endoscopy should be considered as the first-line treatment of TFV due to its superior efficacy, durability, and similar safety, compared to shunt placement and its minimally invasive nature. Microsurgery should be considered as a second treatment option, due to its similar clinical outcomes and revision rate as endoscopy, but it is more invasive in nature.

**Supplementary Information:**

The online version contains supplementary material available at 10.1007/s10143-023-01957-x.

## Introduction

Trapped or isolated fourth ventricle (TFV) is a relatively rare but critical condition, which usually occurs in patients who had a history of intraventricular hemorrhage, inflammation or infection, tumor removal, and ventricular shunt placement and is heralded by delayed symptomatology after a period of relative improvement [1]. The pathophysiology of TFV is based on the arachnoidal blockage of the in and outlets of the fourth ventricle, the aqueduct of Sylvius, and the foramina of Magendie and Luschka, respectively [2]. Therefore, the accumulation of cerebrospinal fluid (CSF) compresses the cerebellum and eventually the brain stem, leading to a mixed cerebellar and brainstem syndrome with potentially fatal consequences, especially if the compression affects the lower medulla [3].

Treatment options consist of shunt placement, endoscopic and microsurgical fenestration. By far, the most common treatment is the placement of a fourth ventricle shunt [4]. However, this procedure carries several risks, including infection, malposition, malfunction, and brain stem lesion [5]. In recent years, given the evolution of endoscopic approaches, several papers have been reporting cases of patients treated by endoscopic aqueductoplasty, either with or without stent placement [6]. The purpose of these techniques is to reestablish the communication between the TFVs, and the third ventricle or the subarachnoid space, obviating the need for a fourth ventricle shunt. Microsurgical fenestration of the fourth ventricle is more invasive and carries risks associated with posterior fossa craniotomies, especially in infants [7].

The purpose of this article is to review all available data on endoscopic, microsurgical, and shunt placement approaches to the treatment of TFV and to perform an individual patient data meta-analysis (IPD) in order to compare the safety, efficacy, and durability of these techniques.

## Methods

### Search strategy and data extraction

With the help of an information specialist from the Erasmus MC Medical Library, Embase, Google Scholar, Medline ALL, Web of Science Core Collection, and Cochrane Central Register of Controlled Trials were searched for articles from inception to September 13, 2022. For the database searches, synonyms to “trapped fourth ventricle,” such as “isolated” and solitaire” were used (Supplementary Appendix: Search strategy).

In order to be included, articles had to contain clinical data on patients diagnosed with and treated for TFV. The reasons for exclusion of articles included: non-English reports and non-original articles, and no TFV patients were included in the study.

Articles identified from database searches were exported to EndNote (Clarivate Analytics). First, the title and abstract of the obtained articles were screened for eligibility by 3 authors (YS, CS, PS). Full-text articles were obtained if their abstracts were considered to be eligible. Each full-text article was then assessed for final inclusion in this systematic review. Disagreements were resolved through discussion with the senior author (VV). Authors were contacted to provide the individual data of their patients. The data from the included articles were divided into two groups, one in which individual patient data was available and one containing aggregate data of reports of individual cohorts.

### Outcome measures and definitions

For the IPD, relevant clinical data on safety (peri- and post-procedural complications and mortality), efficacy, (clinical and radiological improvement) and durability (revision rate, revision technique, and (exact) time until revision) of the interventions were extracted from the included articles. Patient demographics: age, gender, etiology of TFV, clinical presentation, surgical history, and diagnostic imaging techniques were extracted.

All used primary interventions are divided into four main intervention groups: endoscopy, microsurgery, shunt, and hybrid group. The hybrid group consists of TFV patients who had a combination of two or more interventions as their primary intervention.

Peri-procedural complications were defined as intervention-related complications, occurring during surgery and post-procedural complications as intervention-related but occurring after the procedure. This study only contains previously published data in accordance with the Declaration of Helsinki. All patients included in this systematic review have given informed consent in the articles where their data originates from.

Clinical improvement was defined as slight to complete recovery of the initial symptoms. Radiological improvement was defined as a slight to complete reduction in the size of the fourth ventricle, determined by magnetic resonance imaging (MRI) or computer tomography (CT). The time until revision was defined as the period of time elapsed between the primary intervention and the intervention performed as the first revision. This outcome in turn was divided into three categories: early revision (< 3 months), intermediate revision (3–12 months), and late revision (> 12 months).

For the cohort meta-analysis, the same clinical data as stated above were extracted from the included articles. The outcomes on revisions of the IPD were included in the cohort meta-analysis.

### Statistical analysis

Descriptive statistics were used to characterize the baseline demographics of the TFV patients. Normally distributed variables were reported as means and standard deviations (*SD*). Non-normally distributed variables were reported as medians and interquartile ranges (*IQR*). Categorical variables were reported as absolute numbers (*N*) and percentages of the total.

Intervention groups were compared using the chi-squared test for qualitative parameters. Multivariate logistic regression with a random intercept for cohort was used to estimate adjusted odds ratios (*aOR*) and to adjust for confounders. Kaplan-Meier survival curves were generated, and the different techniques were compared using the log-rank test to compare the different techniques in terms of time to revision. The descriptive results of the infant population (< 1 year) were included in the Supplementary Appendix. Statistical analyses were performed using SPSS software (IBM SPSS Statistics for Windows, Version 28.0.1.0. [142], Armonk, New York). A *p*-value < 0.05 was considered as statistically significant, a confidence interval of 95% is used (95% *CI*). The cohort meta-analysis was performed using R, version 4.0.5 (meta package).

## Results

### Study characteristics

**Fig. 1:**
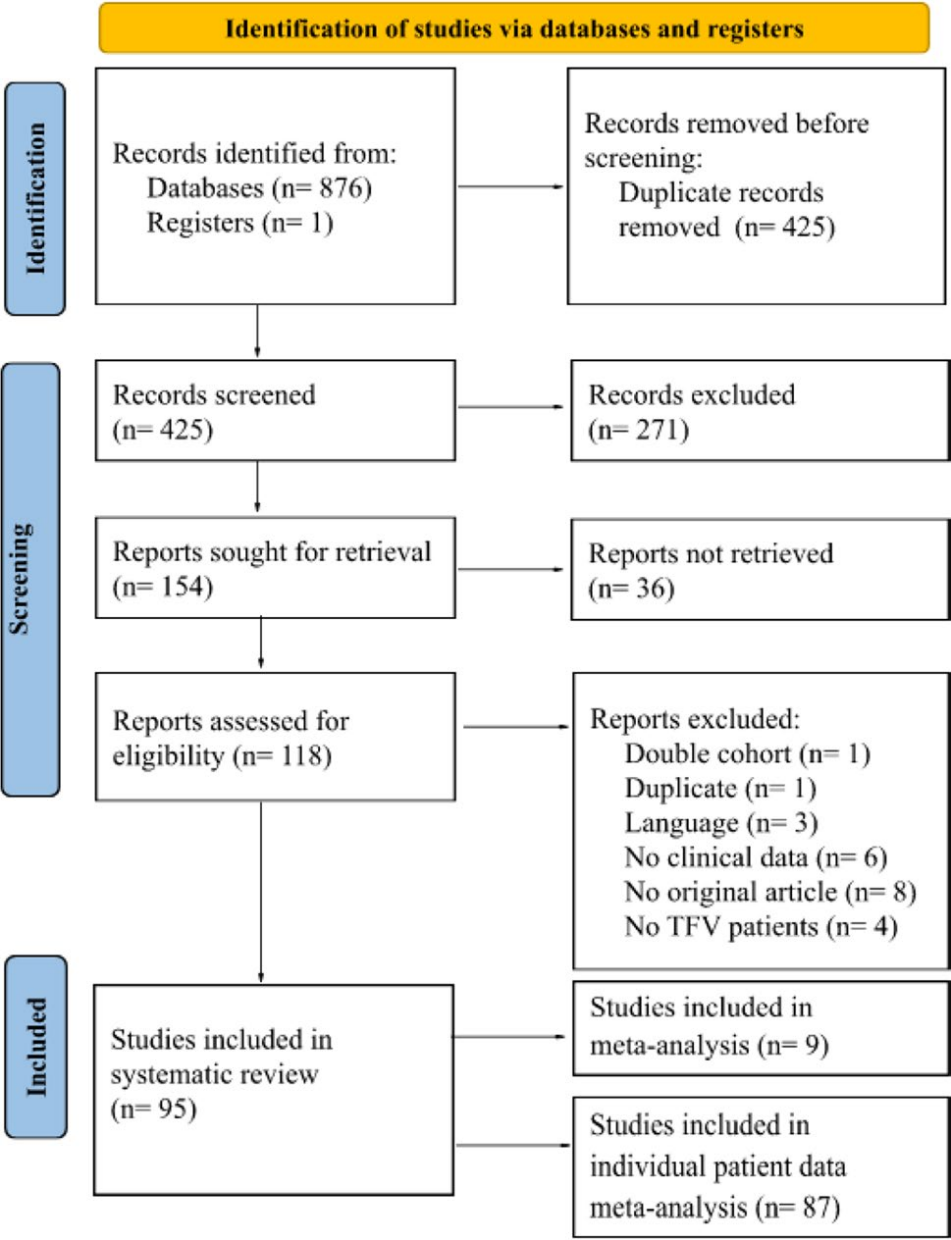
A flowchart of the study selection process [8]

The search strategy yielded 877 articles put through initial screening, of which 425 articles remained after removing duplicates. Two hundred seventy-one articles were excluded based on title and abstract. One hundred fifty-four articles remained to be assessed for eligibility based on full-text sifting. Ultimately, 87 and 9 articles were eligible for the IPD and the cohort meta-analysis, respectively (Fig. 1) (8). A total of 465 patients were included in the IPD and the cohort meta-analysis combined.

Twenty-eight retrospective cohort studies, 22 case series, and 37 case reports were included in the IPD (Supplementary Appendix: Table 3, Table 4a). A total of 314 patients were treated for a TFV with either an endoscopic, microsurgical, shunt placement or a hybrid approach.

In the cohort meta-analysis, 8 retrospective cohort studies and 1 case series were included (Supplementary Appendix: Table 4b).

### Individual patient data meta-analysis

#### Baseline characteristics

The clinical data of a total of 328 patients diagnosed with a TFV were extracted from the included articles. Fourteen TFV patients were excluded from the IPD as they were not treated with either of the four described interventions. These patients were treated conservatively. A total of 314 patients were included in the final analysis. A total of 143 (45%) patients were treated with an endoscopic approach, 45 (14%) with a microsurgical approach, 90 (29%) with a shunt placement approach, and 36 (11%) with a hybrid approach (Table 1). Thirty-two (36%) patients had a shunt placed via infratentorial approaches (Supplementary Appendix: Table 5). The median age was 3 years [*IQR* 1–8] in the endoscopic group, 4 years [*IQR* 1–22.5] in the microsurgical group, 10 years [*IQR* 0–33.5] in the shunt group, and 8.5 years [*IQR* 1–25] in the hybrid group. The etiology of the hydrocephalus in most patients was post-hemorrhagic (*n* = 73, [54%] in the endoscopic group, *n* = 25, [58%] in the microsurgical group, *n* = 20, [25%] in the shunt group, and *n* = 7, [22%] in the hybrid group) (Table 1). Most patients (*n* = 105, [87%] in the endoscopic group, *n* = 43, [96%] in the microsurgical group, *n* = 67, [84%] in the shunt group, and *n* = 22, [85%] in the hybrid group) presented with brain stem signs (Table 1). Most TFV cases (*n* = 108, [85%] in the endoscopic group, *n* = 39, [87%] in the microsurgical group, *n* = 42, [48%] in the shunt group, and *n* = 14, [54%] in the hybrid group) were diagnosed through MRI (Table 1).

**Table 1:** Baseline demographics trapped fourth ventricle patients

	No. (%)					
	All	Endoscopy	Microsurgery	Shunt	Hybrid	*p* value
	(*n* = 314)	(*n* = 143)	(*n* = 45)	(*n* = 90)	(*n* = 36)	
Gender, male	130 (50)	64 (55)	14 (44)	42 (49)	10 (60)	0.53
Age (year), median (*IQR*)	5 (1–17.8)	3 (1–8)	4 (1–22.5)	10 (0–33.5)	8.5 (1–25)	< 0.001
Previous endoscopy	18 (7)	5 (4)	0 (0)	11 (13)	2 (7)	0.005
Previous microsurgery	24 (9)	4 (3)	3 (7)	11 (13)	6 (21)	0.014
Previous shunt placement	247 (90)	107 (93)	41 (91)	73 (85)	26 (93)	0.42
Etiology PHH	125 (43)	73 (54)	25 (58)	20 (25)	7 (22)	< 0.001
Etiology PIH	109 (38)	60 (44)	14 (33)	20 (25)	15 (47)	0.024
Etiology PTH	22 (8)	12 (9)	0 (0)	8 (10)	2 (6)	0.57
Etiology PIC	85 (29)	28 (21)	5 (12)	40 (51)	12(37)	< 0.001
Prematurity	64 (94)	16 (94)	21 (95)	21 (95)	6 (86)	0.72
Clinical presentation						
Cerebellar signs	90 (33)	35 (29)	17 (38)	31 (39)	7 (27)	0.48
Brainstem signs	237 (87)	105 (87)	43 (96)	67 (84)	22 (85)	0.40
Motor dysfunction	73 (23)	24 (20)	14 (31)	27 (35)	8 (36)	0.07
MRI confirmation	203 (71)	108 (85)	39 (87)	42 (48)	14 (54)	< 0.001
CT confirmation	170 (59)	68 (53)	20 (45)	63 (72)	19 (68)	0.008
Clinical improvement						0.008
Yes	272 (95)	126 (98)	44 (98)	75 (90)	27 (90)	
No	15 (5)	3 (2)	1 (2)	8 (10)	3 (10)	
Clinical FU (months), median (*IQR*)	22 (12–44.8)	19 (12–49)	35 (17.5–54.8)	12 (6–24)	30 (18–40)	0.55
Radiological improvement						0.33
Yes	178 (82)	84 (78)	27 (79)	49 (92)	19 (86)	
No	39 (18)	23 (21)	7 (21)	5 (9)	3 (14)	
Radiological FU (months), median (*IQR*)	12 (1–30.5)	8 (1–12)	37.5 (13.5–64.5)	11 (1–24)	23 (6–42)	0.027
Periprocedural complications	8 (6)	3 (7)	0 (0)	3 (6)	2 (12)	0.42
Post-procedural complications	37 (22)	17 (25)	1 (3)	14 (28)	5 (29)	0.037
Mortality	4 (1)	0 (0)	1 (2)	2 (2)	1 (3)	-

*IQR* interquartile range, *PHH* post-hemorrhagic hydrocephalus, *PIH* post-infectious hydrocephalus, *PTH* post-tumorous hydrocephalus, *PIC* post-intervention complication, *MRI* magnetic resonance imaging, *CT* computed tomography, *FU* follow-up

#### Safety outcomes

Peri-procedural complications had an overall incidence of 6% (*n* = 8) for all patients. The overall peri-procedural complications between all interventions yielded no significant difference (*p* = 0.42) (Table 1).

Post-procedural complications had an overall incidence of 22% (*n* = 37) for all patients. The highest rate of post-procedural complications was observed in the hybrid group (*n* = 5; 29%) and the lowest in the microsurgical group (*n* = 1; 3%). The rates of post-procedural complications in the endoscopic and shunt groups were 25% (*n* = 17) and 28% (*n* = 14), respectively. The overall post-procedural complications yielded a significant difference between all interventions (*p* = 0.037) (Table 1). The peri-procedural and post-procedural complications yielded no significant difference between trans-cerebellar and trans-foraminal Magendie approaches in shunt placement (*p* = 0.35, *p* = 0.26) (Supplementary Appendix: Table 5). As for endoscopy, the peri-procedural and post-procedural complications yielded no significant difference between supratentorial and infratentorial approaches (*p* = 0.58, *p* = 0.49) (Supplementary Appendix: Table 6).

Mortality due to the procedure occurred in 1% of all patients (*n* = 4). The overall mortality rates between all interventions yielded no significant difference (Table 1).

#### Effectiveness outcomes

##### Clinical outcomes

The median clinical follow-up was 22 months [*IQR* 12–44.8] for all patients. The microsurgery group had the longest median clinical follow-up of 35 months [*IQR* 17.5–54.8]. Median clinical follow-up was 19 months [*IQR* 12–49] for the endoscopic, 12 months [*IQR* 6–24] for the shunt and 30 months [*IQR* 18–40] for the hybrid group (Table 1).

Clinical improvement was observed in 272 cases (95%) in all patients. The highest rates of clinical improvement were observed in the microsurgery group (*n* = 44; 98%) and in the endoscopy group (*n* = 126; 98%). The shunt group (*n* = 75; 90%) and the hybrid group (*n* = 27; 90%) had a lower rate of clinical improvement. The overall rate of clinical improvement yielded a significant difference between the interventions (*p* = 0.008) (Table 1).

The clinical improvement yielded no significant difference between trans-cerebellar and trans-foraminal Magendie approaches in shunt placement (*p* = 0.89) (Supplementary Appendix: Table 5). For endoscopy, the clinical improvement yielded no significant difference between supratentorial and infratentorial approaches (*p* = 0.95) (Supplementary Appendix: Table 6).

##### Radiological outcomes

The median radiological follow-up was 12 months [*IQR* 1–30.5] for all patients (Table 1).

Radiological improvement was observed in 178 cases (82%) in all patients. Rates of radiological improvement were 84 (78%) in the endoscopy, 27 (79%) in the microsurgery, 49 (92%) in the shunt and 19 (86%) in the hybrid group. The overall rate of radiological improvement yielded no significant difference between the interventions (*p* = 0.33) (Table 1).

The radiological improvement yielded no significant difference between trans-cerebellar and trans-foraminal Magendie approaches in shunt placement (*p* = 0.43) (Supplementary Appendix: Table 5). For endoscopy, the radiological improvement was significantly higher in the supratentorial group (92%) than in the infratentorial group (41%, *p* < 0.001) (Supplementary Appendix: Table 6). Nevertheless, due to potential confounding by indication we cannot make firm statements about these findings.

#### Durability outcomes

A revision was required in 71 patients (38%). The highest revision rate was observed in the shunt group (*n* = 36; 54%). The overall rate of revision yielded a significant difference between the interventions (*p* = 0.003) (Table 2). The revision rate yielded no significant difference between trans-cerebellar and trans-foraminal Magendie approaches in shunt placement (*p* = 0.41) (Supplementary Appendix: Table 5). For endoscopy, the revision rate yielded no significant difference between supratentorial and infratentorial approaches (*p* = 0.59) (Supplementary Appendix: Table 6).

**Table 2:** Revision characteristics

	No. (%)					
	All	Endoscopy	Microsurgery	Shunt	Hybrid	*p* value
	(*n* = 314)	(*n* = 143)	(*n* = 45)	(*n* = 90)	(*n* = 36)	
Revision (yes)	71 (38)	16 (23)	6 (29)	36 (54)	13 (46)	0.003
Revision (*n*), median (*IQR*)	1 (1–2)	1 (1–2)	1 (1–1)	1 (1–2)	1 (1–2.75)	0.67
Revision technique						0.80
Endoscopy	20 (28)	4 (25)	3 (50)	11 (31)	2 (15)	
Microsurgery	3 (4)	0 (0)	1 (17)	2 (6)	0 (0)	
Shunt	34 (48)	7 (44)	1 (17)	18 (50)	8 (61)	
Hybrid	14 (20)	5 (31)	1 (17)	5 (14)	3 (23)	
Time until revision						0.80
Early (< 3 mo)	23 (48)	8 (61)	2 (33)	10 (53)	3 (30)	
Intermediate (3–12 mo)	20 (42)	4 (31)	3 (50)	7 (37)	6 (60)	
Late (> 12 mo)	5 (10)	1 (8)	1 (17)	2 (10)	1 (10)	

*IQR* interquartile range, *mo* months

**Fig. 2:**
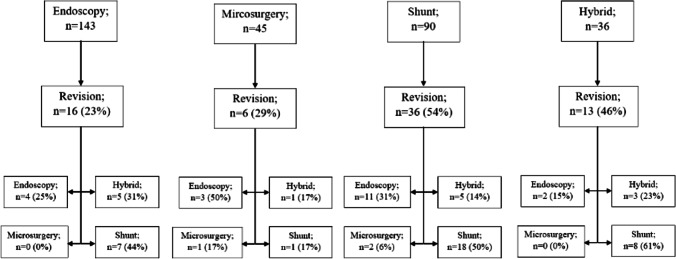
A flowchart of the primary interventions and revisions

The most frequently used revision technique was shunt placement (*n* = 34; 48%) in all cases of revision. The revision rates and techniques employed for each primary intervention are shown in Fig. 2.

For all patients, most revisions were performed in the early (*n* = 23; 48%) and intermediate (*n* = 20; 42%) post-operative period. Only 5 (10%) revisions were performed in the late post-operative period. Between all interventions no significant difference in time until revision was found (*p* = 0.8) (Table 2).

**Fig. 3:**
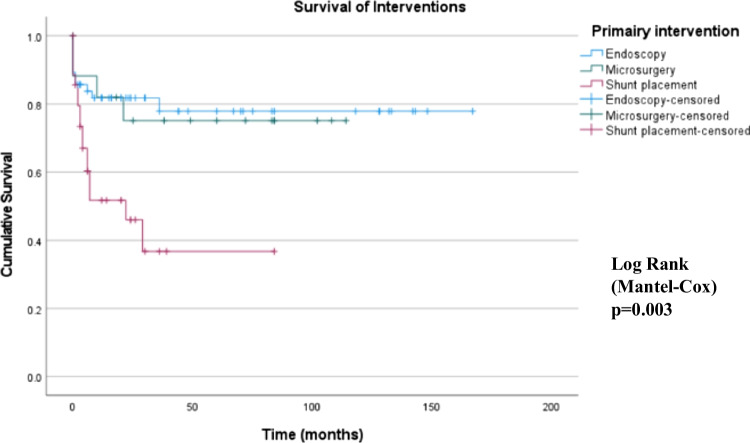
Kaplan-Meier curve, survival of the interventions

Kaplan-Meier analysis revealed that, among the 3 interventions, shunt placement was associated with the worst survival of the primary intervention technique over time (*p* = 0.003; log rank test; Fig. 3). No statistically significant difference in primary intervention survival was found between microsurgery and endoscopy as a primary intervention (*p* = 0.84).

#### Confounder adjustment

TFV patients presenting with cerebellar signs were associated with a significantly lower revision rate (*aOR* 0.38; [95% *CI* 0.15–0.99]) (Table 3). Primary endoscopic (*aOR* 0.21; [95% *CI* 0.07–0.57]) and microsurgical interventions (*aOR* 0.21; [95% *CI* 0.05–0.82]) were also associated with a significantly lower revision rate. Endoscopy was also associated with a significantly higher rate of clinical improvement after confounder adjustment, when compared to shunt placement (*aOR* 4.56; [95% *CI* 1.2–18]).

**Table 3:** Regression analysis all patients (*n* = 314)

	Revision		Clinical improvement	
	*aOR*	95% *CI*	*aOR*	95% *CI*
Gender	-	-	0.31	0.08–1.2
Previous endoscopy	0.93	0.14–6.4	-	-
Previous microsurgery	0.37	0.09–1.5	-	-
Previous shunt placement	0.57	0.57–3.1	-	-
Etiology PHH	2.61	0.97–7.0	-	-
Cerebellar signs	0.38	0.15–0.99	-	-
Intervention: endoscopy	0.21	0.08–0.57	4.56	1.2–18
Intervention: microsurgery	0.21	0.05–0.82	3.33	0.40–28.1

*PHH* post-hemorrhagic hydrocephalus, *aOR* adjusted odds ratio, *CI* confidence interval

#### Cohort meta-analysis

**Fig. 4:**
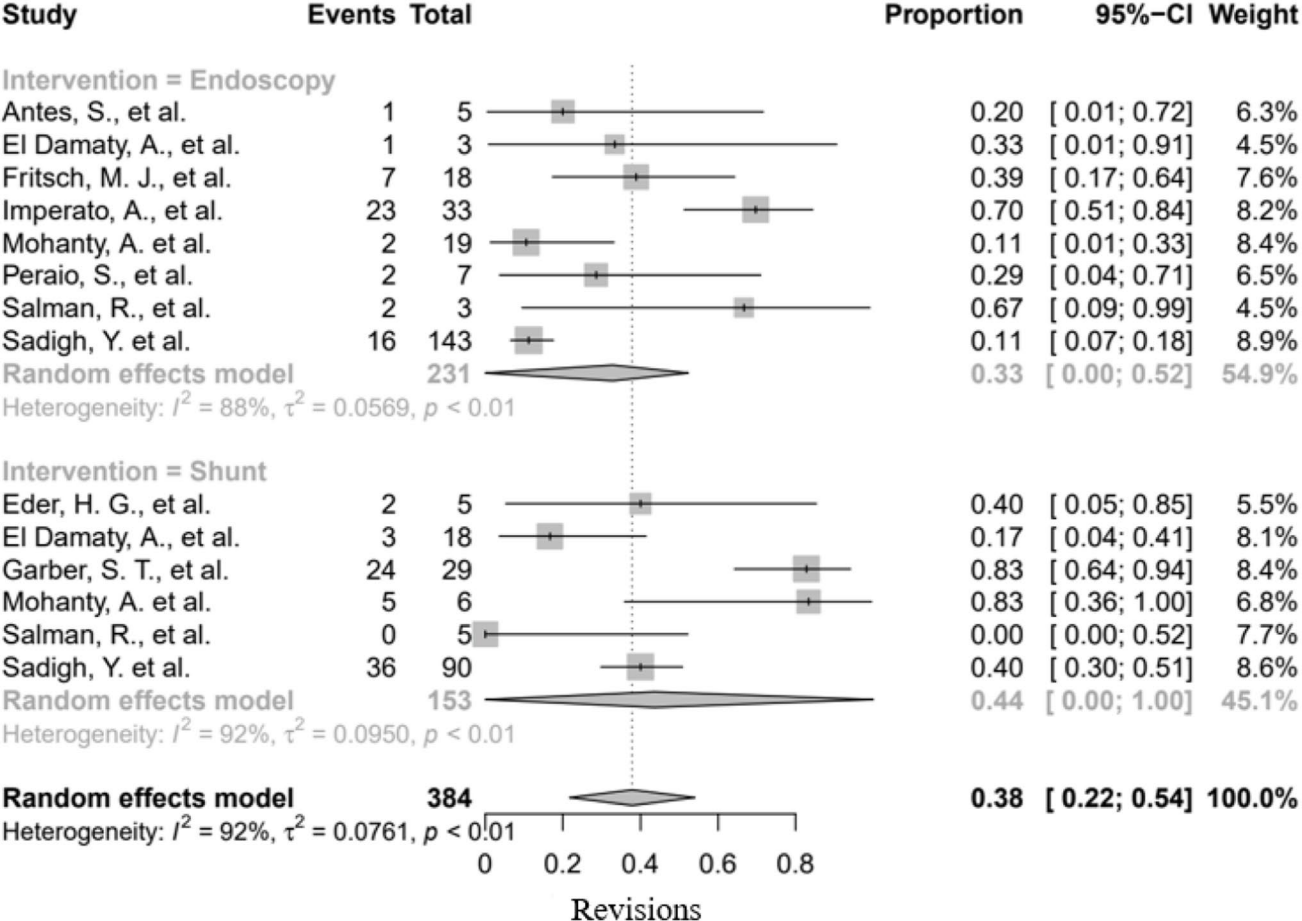
Forest plot revision

In the studies included in the meta-analysis (*n* = 9), a total of 151 patients were treated for a TFV with either an endoscopic or a shunt placement approach. In addition, the endoscopic (*n* = 143) and shunt (*n* = 90) groups of the IPD were included in the meta-analysis. In total, 384 patients were included in the meta-analysis. No significant difference was found between endoscopy and shunt placement in terms of the revision rate. The confidence intervals were very wide due to the scarcity of data, and there was a copious amount of missing data (Fig. 4).

## Discussion

### Summary of findings

This study summarized evidence from 95 articles, including 465 patients. An IPD was performed to evaluate safety, efficacy, and durability of each intervention. A meta-analysis was performed on non-individual patient data from cohorts with the addition of the results of the IPD. The results of the IPD for endoscopy showed a significantly higher clinical improvement, a higher durability, and a lower revision rate, compared to shunt placement. There was no significant difference in safety outcomes between the interventions. The results of the cohort meta-analysis revealed no significant difference in revision rate between endoscopy and shunt placement, but the wide confidence intervals preclude any reasonable conclusion from being drawn.

### Treatment modalities and potential biases

**Fig. 5:**
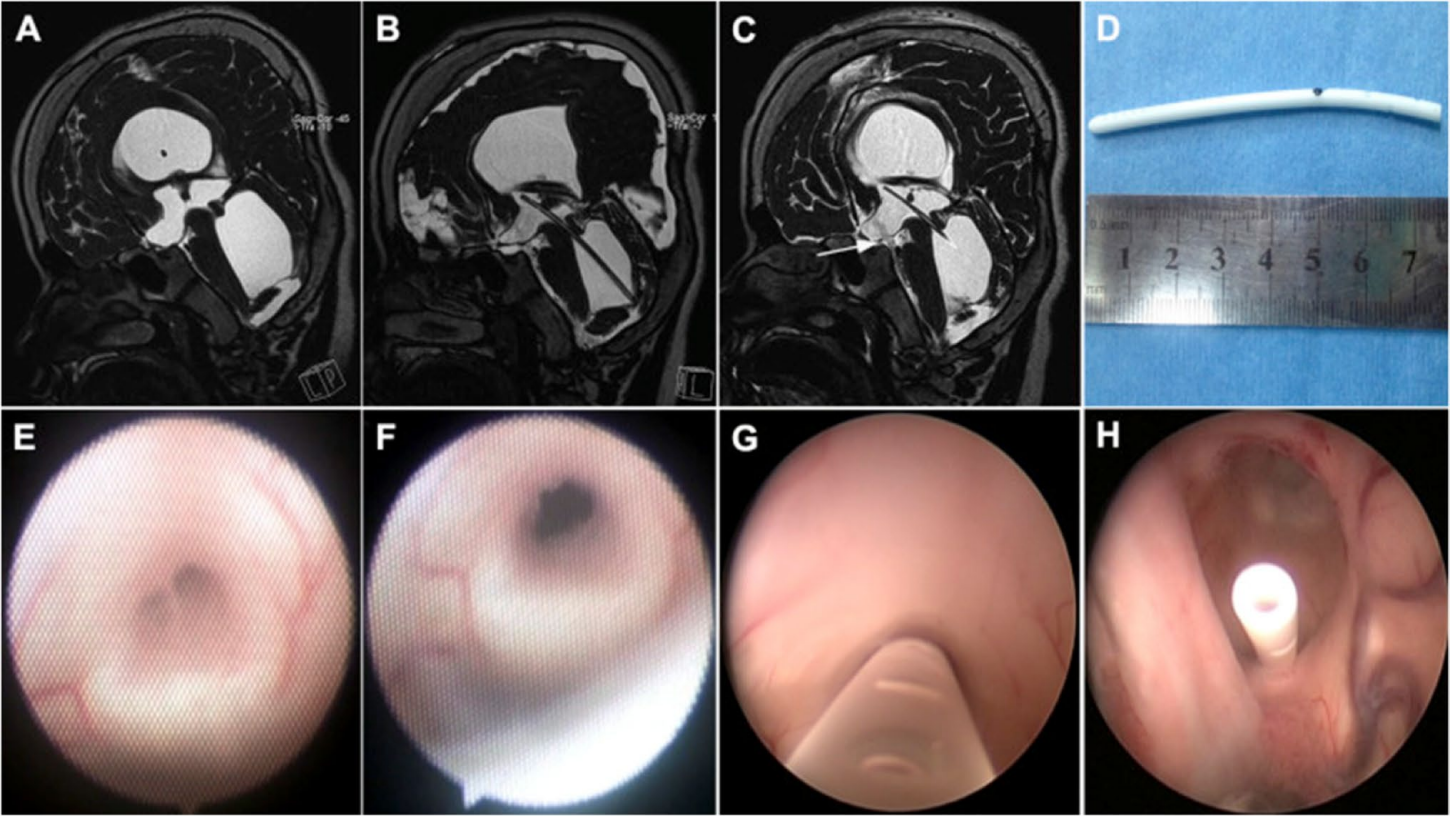
Example of aqueductal stenting (reproduced from Geng et al. [11] with permission). Preoperative sagittal (**A**) magnetic resonance imaging (MRI) demonstrates the aqueduct obstruction associated the large trapped fourth ventricle. Post-operative MRI scans confirmed the stent in good position (**B**) and flow void signal (white arrow) through the anterior floor of the third ventricle (**C**). The tailored aqueduct stent is shown (**D**). Intraoperative findings with the flexible scope revealed membranous aqueductal stenosis (**E**), and patency after aqueductoplasty (**F**). After aqueduct stent placement, the rigid scope was used to check the surface of the catheter to ensure the presence of side hole into the third ventricle (**G**). Final inspection confirmed that the stent coming from the aqueduct through the foramen of Monro into the lateral ventricle (**H**)

In this study, the different treatment modalities for TFV described in the literature were divided into four groups. The applied modifications of these techniques in the four groups might influence the safety, efficacy, and durability of these techniques (e.g., aqueductoplasty and stenting; Fig. 5). As a study by Fritsch et al. [9], which treated a group of 18 TFV patients with either endoscopic aqueductoplasty alone or together with stenting, concluded that patients with a trapped fourth ventricle have a significant restenosis rate following aqueductoplasty. Therefore, initial stent placement is recommended by this study. As for shunt placement, a study by El Damaty et al. [10] mentioned a decline in the occurrence of TFV over the last years of the follow-up, after the introduction of the anti-siphon device in ventriculoperitoneal shunt systems due to the marked decrease in overdrainage. As for the surgical approaches for the shunt placement, no conclusions can be drawn about their effect on the clinical outcomes of TFV patients because of a lack of data reported by the included studies. Future research should look into the possible benefits or indications for modifications of the used treatment techniques and different surgical approaches described in the current study.

No risk of bias assessment was performed in this study. Since TFV has a low prevalence, we aimed to include all evidence available on the different treatment modalities of TFV, making the risk of bias assessment of less relevance. The expected quality of the articles was low due to the rarity of this condition. Thus, the results of the IPD and the meta-analysis should be interpreted with caution. Nevertheless, this is the most comprehensive analysis to date, and the results are robust.

The meta-analysis revealed no significant difference in revision rate of endoscopy, compared to shunt placement. Factors that might have contributed to this finding were wide *CI*s and the probability of publication bias of the included studies, as it is likely that only high-volume centers with considerable expertise would want to present relevant “successful” cases.

### Recommendations

Based on our results, endoscopy should be considered as the first treatment modality due to its lower revision rate and significantly higher overall clinical improvement, compared to shunt placement. Due to its similar clinical outcomes and revision rate as endoscopy but a more invasive nature, microsurgery should be considered as a second treatment option. The outcomes on safety of the microsurgical approach should be interpreted with caution because of a high amount of missing data in this group. Shunt placement could be considered as a last resort in the treatment of TFV, as it is sensitive for severe complications and showed a higher rate of revision, compared to microsurgery and endoscopy.

No recommendations could be made about the hybrid group, due to the vast amount of different treatment combinations included and the small populations within these subgroups.

Endoscopy could even be considered as the first treatment modality in the infant (< 1 year old) population, due to its minimally invasive nature and non-inferiority to the other treatment options (Supplementary Appendix: Table 1 and Table 2). Recommendations in regard to the infant population should be interpreted with caution due to the small study population. More research should be conducted on this topic to confirm these outcomes.

This study revealed no clear contra-indications for treating TFV patients with endoscopy; however, some studies suggest that endoscopy should primarily be performed in TFV patients with a short-segment aqueductal stenosis (< 5 mm) [12–15]. Endoscopic intervention for TFV patients with long-segment aqueductal stenosis (> 5 mm) is potentially unsafe, because of a high risk of upper brain stem damage [15]. In most of the articles included in this systematic review, however, no data on the extent of aqueductal stenosis was reported in patients receiving endoscopic stenting. Future research should look into the possibilities of endoscopy for treating TFV patients, pre-selected based on the extent of aqueductal stenosis.

### Strengths and limitations

To our knowledge, this systematic review included the largest pooled data of TFV patients in an IPD to date. Current data on the treatment of TFV are mostly reported in case reports, case series, and small retrospective cohort studies where few comparisons of the safety, efficacy, and durability of the different treatment modalities are being made.

As expected for retrospective studies, considerable amounts of missing data were encountered, that was no longer retrievable. For example, the microsurgical group contained significant amounts of missing data regarding the outcomes’ safety and durability, which makes it difficult to draw conclusions from the cumulative survival and the safety profile of this method.

Furthermore, clinical data across the included studies was highly heterogeneous in terms of description. For example, outcome measures such as clinical improvement were rarely reported based on a commonly known score scale, such as Modified Rankin Score (mRS) or Glasgow Outcome Scale (GOS), but rather as observations of the patient’s condition. Last but not least, because of the low prevalence of the disease and the expertise required, only centers with sufficient endoscopic surgery expertise should attempt the techniques described in these papers.

## Conclusion

Endoscopy should be considered as the first-line treatment of TFV due to its superior efficacy, durability, and similar safety, compared to shunt placement and its minimally invasive nature. Microsurgery should be considered as a second treatment option, due to its similar clinical outcomes and revision rate as endoscopy, but it is more invasive in nature.

### Supplementary information

ESM 1 (DOCX 55 kb)
