# Supplementary material for: Trapped fourth ventricle: to stent, shunt, or fenestrate—a systematic review and individual patient data meta-analysis
Source: Neurosurg Rev. 2023 Jan 28;46(1):45. doi: 10.1007/s10143-023-01957-x (PMC9884256; doi:10.1007/s10143-023-01957-x)
Supplement: Supplementary file 1 — (DOCX 55 kb) [file 10143_2023_1957_MOESM1_ESM.docx]

**Supplementary Appendix**

**Supplementary Appendix: Search strategy**

Embase.com

- ('trapped fourth ventricle'/de OR 'isolated fourth ventricle'/de OR 'trapped brain fourth ventricle'/de OR 'trapped fourth brain ventricle'/de OR 'isolated brain fourth ventricle'/de OR (('brain fourth ventricle'/de OR (((fourth OR 4th OR 4-th OR quart* OR IV) NEXT/6 ventric*)):ab,ti) AND (((trapped OR isolated OR solitar*) NEAR/6 ventric*):ab,ti)))

Medline ALL Ovid

- (Fourth Ventricle/ OR (((fourth OR 4th OR 4-th OR quart* OR IV) ADJ6 ventric*)).ab,ti.) AND (((trapped OR isolated OR solitar*) ADJ6 ventric*).ab,ti.)

Web of science

- TS=((((fourth OR 4th OR 4-th OR quart* OR IV) NEAR/5 ventric*)) AND ((trapped OR isolated OR solitar*) NEAR/5 ventric*))

Cochrane CENTRAL

- (((fourth OR 4th OR "4 th" OR quart* OR IV) NEXT/6 ventric*)):ab,ti AND ((trapped OR isolated OR solitar*) NEAR/6 ventric*):ab,ti

| **Supplementary Appendix: Table 1. Baseline Demographics Trapped fourth ventricle patients (<1 year old)** | | | | | | | |
| --- | --- | --- | --- | --- | --- | --- | --- |
|  | | **No. (%)** | | | | | |
|  |  | **All** | **Endoscopy** | **Microsurgery** | **Shunt** | **Hybrid** | **P Value** |
|  |  | **(n=61)** | **(n=27)** | **(n=5)** | **(n=23)** | **(n=6)** |  |
| Gender, male | | 26 (63) | 9 (60) | 1 (100) | 15 (71) | 1 (25) | 0.43 |
| Age (months), median (IQR) | | 5 (1-7) | 5.5 (3-8.7) | 8 (5.5-10.5) | 1 (0-5) | 7.5 (5.5-8.8) | 0.10 |
| Previous endoscopy | | 1 (2) | 0 (0) | 0 (0) | 1 (4) | 0 (0) | 0.96 |
| Previous microsurgery | | 2 (3) | 0 (0) | 0 (0) | 1 (4) | 1 (20) | <0.001 |
| Previous shunt placement | | 54 (95) | 21 (87) | 5 (100) | 23 (100) | 5 (100) | 0.63 |
| Etiology PHH | | 38 (68) | 18 (78) | 4 (80) | 13 (56) | 3 (60) | 0.40 |
| Etiology PIH | | 22 (39) | 15 (65) | 2 (40) | 2 (9) | 3 (60) | 0.002 |
| Etiology PTH | | 1 (2) | 0 (0) | 0 (0) | 1 (4) | 0 (0) | 0.96 |
| Etiology PIC | | 11 (20) | 3 (13) | 0 (0) | 6 (26) | 2 (40) | 0.08 |
| Prematurity | | 19 (90) | 5 (83) | 3 (75) | 9 (100) | 2 (100) | 0.62 |
| Clinical presentation | | | | | | |  |
| Cerebellar signs | | 8 (16) | 2 (10) | 0 (0) | 6 (32) | 0 (0) | 0.50 |
| Brainstem signs | | 35 (71) | 15 (75) | 5 (100) | 12 (63) | 3 (60) | 0.18 |
| Motor dysfunction | | 17 (35) | 4 (20) | 1 (20) | 11 (58) | 1 (20) | 0.09 |
| MRI confirmation | | 24 (47) | 16 (80) | 4 (80) | 2 (9) | 2 (50) | <0.001 |
| CT confirmation | | 38 (69) | 10 (43) | 4 (80) | 20 (91) | 4 (80) | 0.01 |
| Clinical improvement | | | | | | | 0.46 |
|  | Yes | 50 (94) | 23 (100) | 5 (100) | 16 (84) | 6 (100) |  |
|  | No | 3 (6) | 0 (0) | 0 (0) | 3 (16) | 0 (0) |  |
| Clinical FU (months), median (IQR) | | 25 (7-53) | 19 (8-53) | 33 (28-78) | 6 (0.5-63) | 30.5 (8.5-39.7) | 0.61 |
| Radiological improvement | | | | | | | 0.83 |
|  | Yes | 24 (86) | 14 (87) | 2 (67) | 5 (83) | 3 (100) |  |
|  | No | 4 (14) | 2 (12) | 1 (33) | 1 (17) | 0 (0) |  |
| Radiological FU (months), median (IQR) | | 12 (1-53) | 5.5 (0-30) | 72 (25-72) | 15.5 (1-79.5) | 12.5 (0-12.5) | 0.13 |
| Periprocedural complications | | 3 (13) | 2 (17) | 0 (0) | 0 (0) | 1 (33) | 0.14 |
| Postprocedural complications | | 2 (8) | 2 (14) | 0 (0) | 0 (0) | 0 (0) | 0.87 |
| Mortality | | 1 (2) | 0 (0) | 0 (0) | 0 (0) | 1 (100) | - |

**IQR: Interquartile range, PHH: Post-hemorrhagic hydrocephalus, PIH: Post-infectious hydrocephalus, PTH: Post-tumorous hydrocephalus, PIC: Post-intervention complication, MRI: Magnetic resonance imaging, CT: Computed tomography, FU: Follow-up.**

| **Supplementary Appendix: Table 2. Revision characteristics (patients <1 year old)** | | | | | | | |
| --- | --- | --- | --- | --- | --- | --- | --- |
|  | | **No. (%)** | | | | | |
|  |  | **All** | **Endoscopy** | **Microsurgery** | **Shunt** | **Hybrid** | **P Value** |
|  |  | **(n=61)** | **(n=27)** | **(n=5)** | **(n=23)** | **(n=6)** |  |
| Revision (yes) | | 14 (47) | 5 (42) | 1 (25) | 6 (60) | 2 (50) | 0.23 |
| Revision (n), median (IQR) | | 2 (1-2) | 1 (1-1.5) | 2 (1-1) | 1 (1-1.3) | 2 (1-2) | 0.34 |
| Revision technique | | | | | | | 0.39 |
|  | Endoscopy | 3 (21) | 1 (20) | 1 (100) | 0 (0) | 1 (50) |  |
|  | Microsurgery | 1 (7) | 0 (0) | 0 (0) | 1 (17) | 0 (0) |  |
|  | Shunt | 6 (43) | 2 (40) | 0 (0) | 4 (67) | 0 (0) |  |
|  | Hybrid | 4 (28) | 2 (40) | 0 (0) | 1 (17) | 1 (50) |  |
| Time until revision | | | | | | | 0.75 |
|  | Early (<3 mo) | 3 (23) | 2 (40) | 0 (0) | 1 (20) | 0 (0) |  |
|  | Intermediate (3-12 mo) | 9 (69) | 3 (60) | 1 (100) | 3 (60) | 2 (100) |  |
|  | Late (>12 mo) | 1 (8) | 0 (0) | 0 (0) | 1 (20) | 0 (0) |  |

**IQR: Interquartile range, mo: months.**

| **Supplementary Appendix: Table 3. Baseline characteristics articles (patient individual analysis)** | | | | | |
| --- | --- | --- | --- | --- | --- |
| **Study design** | | **RC** | **CS** | **CR** | **Total** |
| **Publication year** | | 1978-2021 | 1978-2021 | 1982-2021 |  |
| **N studies** | | 28 | 22 | 37 | 87 |
| **N TFV patients** | | 208 | 70 | 36 | 314 |
| **Article origin** | Africa | 2 | 0 | 1 | 3 |
|  | Asia | 10 | 1 | 20 | 31 |
|  | Europe | 7 | 8 | 4 | 19 |
|  | North America | 7 | 10 | 11 | 28 |
|  | Oceania | 0 | 2 | 0 | 2 |
|  | South America | 1 | 1 | 1 | 3 |
|  | NA | 1 | 0 | 0 | 1 |

**TFV: Trapped fourth ventricle, RC: Retrospective cohort study, CS: Case series, CR: Case report.**

| **Supplementary Appendix Table 4a: Study characteristics of studies included in the patient individual analysis** | | | |
| --- | --- | --- | --- |
| **Author** | **Year** | **Study design** | **Country** |
| Ali, K., et al. ^1^ | 2013 | CS | UK |
| Anegawa, S., et al. ^2^ | 1993 | CR | Japan |
| Aoki, N. ^4^ | 1987 | CR | Japan |
| Armbruster, L., et al. ^5^ | 2012 | RC | Germany |
| Banh, L., et al. ^6^ | 2013 | CS | Australia |
| Barami, K., et al. ^7^ | 2018 | CS | USA |
| Bhatia, A., et al. ^8^ | 2016 | CR | USA |
| Carter, L. M., et al. ^9^ | 2021 | CS | USA |
| Cinalli, G., et al. ^10^ | 2006 | CS | Italy |
| Coker, S. B., et al. ^11^ | 1986 | CR | USA |
| Coker, S. B. and C. L. Anderson ^12^ | 1989 | RC | USA |
| Colli, B. O., et al. ^13^ | 1993 | CS | Brazil |
| Colpan, M. E., et al. ^14^ | 2003 | CR | Turkey |
| Dauda, H. A., et al. ^15^ | 2021 | CR | Nigeria |
| Dhiwakar, M., et al. ^16^ | 2004 | CR | UK |
| Dollo, C., et al. ^17^ | 2001 | CS | Italy |
| Edison, P. E., et al. ^19^ | 2020 | CR | Singapore |
| Elgamal, M. E., et al. ^21^ | 2018 | CR | UAE |
| Eller, T. W., et al. ^22^ | 1985 | CS | USA |
| Elsharkawy, A. A., et al. ^23^ | 2020 | RC | Egypt |
| Erşahin, Y. ^24^ | 2007 | RC | Turkey |
| Ersahin, Y., et al. ^25^ | 1992 | CR | Turkey |
| Ferreira, M., et al. ^26^ | 2012 | RC | USA |
| Frassanito, P., et al. ^27^ | 2015 | CR | Italy, Greece |
| Furtado, L. M. F., et al. ^29^ | 2021 | RC | Brazil |
| Gallo, P., et al. ^30^ | 2012 | RC | France |
| Garg, K., et al. ^32^ | 2021 | CR | India |
| Geng, J., et al. ^33^ | 2015 | RC | China |
| Guida, L., et al. ^34^ | 2020 | RC | France |
| Oi, S., et al. ^62^ | 1999 | RC | Japan |
| Ogiwara, H. and N. Morota ^61^ | 2013 | RC | Japan |
| Oi, S. and S. Matsumoto ^63^ | 1985 | RC | Japan |
| Oi, S. and S. Matsumoto ^64^ | 1986 | CS | Japan |
| Otsuki, T., et al. ^65^ | 1982 | CR | Japan |
| Owler, B. K., et al. ^66^ | 2001 | CS | Australia |
| Pang, D., et al. ^67^ | 2005 | CR | USA |
| Raouf, A. and I. Zidan ^69^ | 2013 | RC | Egypt |
| Rosenfeld, D. L., et al. ^70^ | 1995 | CS | USA |
| Sagan, L. M., et al. ^71^ | 2006 | CS | Poland |
| Sansone, J. M. and B. J. Iskandar ^73^ | 2005 | CS | USA |
| Say, I., et al. ^74^ | 2019 | CR | USA |
| Schulz, M., et al. ^76^ | 2013 | RC | Germany |
| Schulz, M., et al. ^77^ | 2012 | RC | Germany |
| Schaumann, A., et al. ^75^ | 2021 | RC | Germany |
| Scotti, G., et al. ^78^ | 1980 | RC | USA |
| Sharma, R. R., et al. ^79^ | 2001 | CR | Oman |
| Shin, M., et al.^80^ | 2000 | CR | Japan |
| Simonin, A., et al. ^81^ | 2015 | CS | Switzerland |
| Spennato, P., et al. ^82^ | 2007 | RC | Italy |
| Spennato, P., et al. ^83^ | 2005 | CS | Italy |
| Thakker, R. and A. Mohanty ^84^ | 2019 | CR | USA |
| Tirado-Caballero, J., et al. ^85^ | 2021 | CS | Spain |
| Torres-Corzo, J., et al. ^86^ | 2004 | RC | Mexico |
| Torrez-Corzo, J., et al. ^87^ | 2009 | CR | Mexico |
| Tseng, J. S., et al. ^88^ | 2007 | CR | Taiwan |
| Tyagi, G., et al. ^89^ | 2020 | RC | India |
| Udayakumaran, S., et al. ^90^ | 2010 | RC | Israel |
| Udayakumaran, S., et al. ^91^ | 2009 | CR | Israel |
| Udayakumaran, S. and D. Panikar ^92^ | 2012 | CR | India |
| Upchurch, K., et al. ^93^ | 2007 | CS | USA |
| Villavicencio, A. T., et al. ^94^ | 1998 | CS | USA |
| Zimmerman, R. A., et al. ^95^ | 1978 | RC | USA |
| Hamada, H., et al. ^35^ | 2005 | RC | Japan |
| Hamada, H., et al. ^36^ | 1999 | CR | Japan |
| Hamada, H., et al. ^37^ | 2004 | CR | Japan |
| Hayashi, N., et al. ^40^ | 2000 | RC | Japan |
| Imperato, A., et al. ^43^ | 2021 | RC | NA |
| Jonathan Pomeraniec, I., et al. ^43^ | 2016 | RC | USA |
| Kamgarpour, A. and B. Malekpour ^44^ | 2006 | CR | Iran |
| Khayat, H. A., et al. ^45^ | 2019 | CR | Canada |
| Kim, H. M. and K. H. Kim ^46^ | 2015 | RC | South Korea |
| Kim, S. H., et al. ^47^ | 2012 | CR | South Korea |
| Kumar, V., et al. ^48^ | 2017 | CR | Brazil |
| Harrison, H. R., et al. ^38^ | 1982 | CR | USA |
| Hawkins Iii, J. C., et al. ^39^ | 1978 | CS | Canada |
| Hubbard, J. L., et al. ^41^ | 1987 | CR | USA |
| Lewis, C. S., et al. ^49^ | 2018 | CR | USA |
| Little, A. S., et al. ^50^ | 2010 | CR | USA |
| Longatti, P., et al. ^51^ | 2013 | CS | Italy |
| Lourie, H., et al. ^52^ | 1980 | CS | USA |
| Madlinger, A. G. and J. K. Krauss ^53^ | 1998 | CR | Germany |
| Morina, D., et al. ^59^ | 2013 | CR | Germany |
| Maramattom, B. and D. Panikar ^54^ | 2016 | CR | India |
| Mohanty, A. ^56^ | 2005 | RC | USA |
| Marianayagam, N. J., et al. ^55^ | 2017 | CR | Israel |
| Montes, J. L., et al. ^58^ | 1994 | CS | Canada |
| Niwa, J., et al. ^60^ | 1992 | CR | Japan |

**RC: Retrospective cohort study, CS: Case series, CR: Case report.**

| **Supplementary Appendix Table 4b: Study characteristics of studies included in the cohort meta-analysis** | | | |
| --- | --- | --- | --- |
| **Author** | **Year** | **Study design** | **Country** |
| Antes, S., et al. ^3^ | 2016 | CS | Germany |
| Eder, H. G., et al. ^18^ | 1997 | RC | USA |
| El Damaty, A., et al. ^20^ | 2020 | RC | Germany, Egypt, Sweden |
| Fritsch, M. J., et al. ^28^ | 2004 | RC | Germany |
| Garber, S. T., et al. ^31^ | 2013 | RC | USA |
| Imperato, A., et al. ^42^ | 2021 | RC | NA |
| Mohanty, A. and K. Manwaring ^57^ | 2018 | RC | USA |
| Peraio, S., et al. ^68^ | 2018 | RC | Italy, Egypt |
| Salman, R, et al. ^72^ | 2022 | RC | USA |

**RC: Retrospective cohort study, CS: Case series.**

| **Supplementary Appendix: Table 5: Surgical approaches for shunt placement** | | | | | |
| --- | --- | --- | --- | --- | --- |
|  | | **No. (%)** | | | |
|  |  | **All** | **Trans-cerebellar** | **Trans-foraminal Magendie** | **P Value** |
|  |  | **(n=32)** | **(n=14)** | **(n=18)** |  |
| Gender, male | | 11 (35) | 5 (36) | 6 (35) | 0.98 |
| Age (years), median (IQR) | | 14 (3-35) | 27 (11-46) | 4 (2-16) | 0.36 |
| Clinical improvement | | | | | 0.89 |
|  | Yes | 29 (93) | 13 (93) | 16 (94) |  |
|  | No | 2 (6) | 1 (7) | 1 (6) |  |
| Radiological improvement | | | | | 0.43 |
|  | Yes | 18 (95) | 7 (100) | 11 (92) |  |
|  | No | 1 (5) | 0 (0) | 1 (8) |  |
| Periprocedural complications | | 3 (15) | 0 (0) | 3 (19) | 0.35 |
| Postprocedural complications | | 7 (32) | 3 (50) | 4 (25) | 0.26 |
| Revision (yes) | | 10 (42) | 4 (33) | 6 (50) | 0.41 |

**IQR: Interquartile range.**

| **Supplementary Appendix: Table 6. Surgical approach for endoscopy** | | | | | |
| --- | --- | --- | --- | --- | --- |
|  | | **No. (%)** | | | |
|  |  | **All** | **Supratentorial** | **Intratentorial** | **P Value** |
|  |  | **(n=128)** | **(n=87)** | **(n=41)** |  |
| Gender, male | | 61 (57) | 41 (60) | 20 (51) | 0.36 |
| Age (years), median (IQR) | | 3 (1-8.5) | 2 (0-7) | 5 (2-9) | 0.15 |
| Clinical improvement | | | | | 0.95 |
|  | Yes | 114 (97) | 74 (97) | 40 (98) |  |
|  | No | 3 (3) | 2 (3) | 1 (2) |  |
| Radiological improvement | | | | | <0.001 |
|  | Yes | 79 (81) | 70 (92) | 9 (41) |  |
|  | No | 19 (19) | 6 (8) | 13 (59) |  |
| Periprocedural complications | | 3 (8) | 3 (9) | 0 (0) | 0.58 |
| Postprocedural complications | | 15 (25) | 10 (28) | 5 (20) | 0.49 |
| Revision (yes) | | 10 (18) | 7 (20) | 3 (14) | 0.59 |

**IQR: Interquartile range.**

**References**

1. Ali, K., et al. (2013). "The isolated fourth ventricle." BMJ Case Rep.
2. Anegawa, S., et al. (1993). "Dilated fourth ventricle in Arnold-Chiari malformation type II: Isolated fourth ventricle as sequelae of shunt? Case report." NEUROL MED -CHIR 33(8): 575-578.
3. Antes, S., et al. (2016). "Aqueductal stenting with an intra-catheter endoscope—a technical note." CHILD'S NERV SYST 32(2): 359-363.
4. Aoki, N. (1987). "Communicating fourth ventricular hydrocephalus: Case report." NEUROSURGERY 20(5): 806-808.
5. Armbruster, L., et al. (2013). "Microsurgical outlet restoration in isolated fourth ventricular hydrocephalus: A single-institutional experience." CHILD'S NERV SYST 29(9): 1714-1715.
6. Banh, L. and B. P. Brophy (2013). "Cranio-cervical decompression and expansile duroplasty for isolated fourth ventricle in a patient with Chiari II malformation." J Clin Neurosci 20(1): 158-161.
7. Barami, K., et al. (2018). "Diagnosis, Classification, and Management of Fourth Ventriculomegaly in Adults: Report of 9 Cases and Literature Review." World Neurosurg. 116: E709-E722.
8. Bhatia, A. and A. N. Pollock (2016). "Trapped fourth ventricle with vasogenic edema." Pediatr Emerg Care 32(1): 58-59.
9. Carter, L. M. and N. L. Gross (2021). "Endoscopic Placement of Fourth Ventricular Catheter Using Seldinger Technique: Description of Technique and Case Series." Oper Neurosurg 21(4): E304-E308.
10. Cinalli, G., et al. (2006). "Endoscopic aqueductoplasty and placement of a stent in the cerebral aqueduct in the management of isolated fourth ventricle in children." J NEUROSURG 104 PEDIATRICS(SUPPL. 1): 21-27.
11. Coker, S. B. (1986). "Bobble-head doll syndrome due to trapped fourth ventricle and aqueduct." PEDIATR NEUROL 2(2): 115-116.
12. Coker, S. B. and C. L. Anderson (1989). "Occluded fourth ventricle after multiple shunt revisions for hydrocephalus." PEDIATRICS 83(6): 981-985.
13. Colli, B. O., et al. (1993). "Isolated fourth ventricle in neurocysticercosis: Pathophysiology, diagnosis, and treatment." SURG NEUROL 39(4): 305-310.
14. Colpan, M. E., et al. (2003). "Stereotactically-guided fourth ventriculo-peritoneal shunting for the isolated fourth ventricle." Minimally Invasive Neurosurg 46(1): 57-60.
15. Dauda, H. A. and D. Sale (2021). "Trapped fourth ventricle: A case report and review of literature." Int J Surg Case Rep 80.
16. Dhiwakar, M., et al. (2004). "Neurolisteriosis causing hydrocephalus, trapped fourth ventricle, hindbrain hemiation and syringomyelia." Br J Neurosurg 18(4): 367-370.
17. Dollo, C., et al. (2001). "Outlet fenestration for isolated fourth ventricle with and without an internal shunt." CHILD'S NERV SYST 17(8): 483-486.
18. Eder, H. G., et al. (1997). "Complications after shunting isolated IV ventricles." CHILD'S NERV SYST 13(1): 13-16.
19. Edison, P. E., et al. (2020). "Early entrapment of fourth ventricle following Pseudomonas meningitis in extreme prematurity: Case report." J Neonatal-Perinat Med 13(4): 581-586.
20. El Damaty, A., et al. (2020). "Trapped fourth ventricle: a rare complication in children after supratentorial CSF shunting." CHILD'S NERV SYST 36(12): 2961-2969.
21. Elgamal, M. E., et al. (2018). "Iatrogenic (Traumatic) Occipital Artery Pseudoaneurysm - Rare Complication of Ventriculoperitoneal Shunt in an Infant: Case Report and Review of the Literature." Asian J Neurosurg 13(3): 914-917.
22. Eller, T. W. and J. F. Pasternak (1985). "Isolated ventricles following intraventricular hemorrhage." J NEUROSURG 62(3): 357-362.
23. Elsharkawy, A. A. and H. Elatrozy (2020). "Endoscopic antegrade aqueductoplasty and stenting with panventricular catheter in management of trapped fourth ventricle in patients with inadequately functioning supratentorial shunt." Surg Neurol Intl 11.
24. Erşahin, Y. (2007). "Endoscopic aqueductoplasty." CHILD'S NERV SYST 23(2): 143-150.
25. Ersahin, Y., et al. (1992). "A Case of Double-Compartment Hydrocephalus Presenting with Opisthotonos." Surg. Neurol. 38(4): 291-293.
26. Ferreira, M., et al. (2012). "Trapped fourth ventricle phenomenon following aneurysm rupture of the posterior circulation: Case reports." NEUROSURGERY 70(1): E253-E258.
27. Frassanito, P., et al. (2015). "Descending transtentorial herniation, a rare complication of the treatment of trapped fourth ventricle: Case report." J Neursurg Pediatr 16(5): 540-544.
28. Fritsch, M. J., et al. (2004). "Endoscopic aqueductoplasty: Stent or not to stent?" CHILD'S NERV SYST 20(3): 137-142.
29. Furtado, L. M. F., et al. (2021). "Proposed radiological score for the evaluation of isolated fourth ventricle treated by endoscopic aqueductoplasty." CHILD'S NERV SYST 37(4): 1103-1111.
30. Gallo, P., et al. (2012). "The endoscopic trans-fourth ventricle aqueductoplasty and stent placement for the treatment of trapped fourth ventricle: Long-term results in a series of 18 consecutive patients." Neurol India 60(3): 271-277.
31. Garber, S. T., et al. (2013). "Comparing fourth ventricle shunt survival after placement via stereotactic transtentorial and suboccipital approaches." J Neursurg Pediatr 11(6): 623-629.
32. Garg, K., et al. (2021). "“Malignant” Craniospinal Neurocysticercosis: A Rare Case." World Neurosurg 146: 95-102.
33. Geng, J., et al. (2015). "Aqueduct Stent Placement: Indications, Technique, and Clinical Experience." World Neurosurg 84(5): 1347-1353.
34. Guida, L., et al. (2020). "Endoscopic aqueductal stenting in the management of pediatric hydrocephalus." J Neursurg Pediatr 26(4): 346-352.
35. Hamada, H., et al. (2005). "Efficacy of a navigation system in neuroendoscopic surgery." Minimally Invasive Neurosurg 48(4): 197-201.
36. Hamada, H., et al. (1999). "Endoscopic aqueductal plasty via the fourth ventricle through the cerebellar hemisphere under navigating system guidance - Technical note." NEUROL MED -CHIR 39(13): 950-954.
37. Hamada, H., et al. (2004). "Isolated third and fourth ventricles associated with neurosarcoidosis successfully treated by neuroendoscopy - Case report." NEUROL MED -CHIR 44(8): 435-437.
38. Harrison, H. R. and A. F. Reynolds (1982). "Trapped fourth ventricle in coccidioidal meningitis." SURG NEUROL 17(3): 197-199.
39. Hawkins Iii, J. C., et al. (1978). "Isolated fourth ventricle as a complication of ventricular shunting. Report of three cases." J NEUROSURG 49(6): 910-913.
40. Hayashi, N., et al. (2000). "Clinical features in patients requiring reoperation after failed endoscopic procedures for hydrocephalus." Minimally Invasive Neurosurg 43(4): 181-186.
41. Hubbard, J. L., et al. (1987). "Trapped fourth ventricle in an adult: Radiographic findings and surgical treatment." SURG NEUROL 28(4): 301-306.
42. Imperato, A., et al. (2021). "Endoscopic aqueductoplasty and stenting in the treatment of isolated fourth ventricle in children: 20-year institutional experience." CHILD'S NERV SYST 37(5): 1587-1596.
43. Jonathan Pomeraniec, I., et al. (2016). "Frequency and long-term follow-up of trapped fourth ventricle following neonatal posthemorrhagic hydrocephalus." J Neursurg Pediatr 17(5): 552-557.
44. Kamgarpour, A. and B. Malekpour (2006). "Cranial nerve involvement as a complication of ventriculoperitoneal shunt: A case report and review." Neurosurg Q 16(3): 147-151.
45. Khayat, H. A., et al. (2019). "Surgical Management of Isolated Fourth Ventricular Hydrocephalus Associated with Injury to the Guillain-Mollaret Triangle." World Neurosurg 122: 71-76.
46. Kim, H. M. and K. H. Kim (2015). "Clinical Experience of Infantile Posthemorrhagic Hydrocephalus Treated with Ventriculo-Peritoneal Shunt." Korean j. neurotrauma 11(2): 106-111.
47. Kim, S. H., et al. (2012). "Treatment of hydrocephalus associated with neurosarcoidosis by multiple shunt placement." J Korean Neurosurg Soc 52(3): 270-272.
48. Kumar, V., et al. (2017). "Trapped fourth ventricle in recurrent acoustic schwannoma - A rarity." Arq Bras Neurocir 36(1): 71-74.
49. Lewis, C. S., et al. (2018). "Feasibility of a Fourth Ventriculopleural Shunt for Diversion of an Isolated Fourth Ventricle: A Technical Note." Asian J Neurosurg 13(3): 897-900.
50. Little, A. S., et al. (2010). "Simplified aqueductal stenting for isolated fourth ventricle using a small-caliber flexible endoscope in a patient with neurococcidiomycosis: technical case report." NEUROSURGERY 66(6 Suppl Operative): 373-374; discussion 374.
51. Longatti, P., et al. (2013). "The marionette technique for treatment of isolated fourth ventricle." J Neursurg Pediatr 12(4): 339-343.
52. Lourie, H., et al. (1980). "Trapped fourth ventricle: A report of two unusual cases." NEUROSURGERY 7(3): 279-282.
53. Madlinger, A. G. and J. K. Krauss (1998). "Intramedullary brain stem cyst and trapped IV ventricle after infection with Listeria monocytogenes." CHILD'S NERV SYST 14(12): 747-750.
54. Maramattom, B. and D. Panikar (2016). "Bilateral abducens and facial nerve palsies as a localizing sign due to reduction in intracranial pressure after fourth ventriculoperitoneal shunting." Ann Indian Acad Neurol 19(4): 510-511.
55. Marianayagam, N. J., et al. (2017). "Paradoxical ventriculomegaly due to low-pressure hydrocephalus, a rare complication of the treatment of a trapped fourth ventricle: Case report." J Clin Neurosci 39: 101-103.
56. Mohanty, A. (2005). "Endoscopic options in the management of isolated fourth ventricles: Case report." J NEUROSURG 103 PEDIATRICS(SUPPL. 1): 73-78.
57. Mohanty, A. and K. Manwaring (2018). "Isolated fourth ventricle: To shunt or stent." Oper Neurosurg 14(5): 483-493.
58. Montes, J. L., et al. (1994). "Stereotactic transtentorial hiatus ventriculoperitoneal shunting for the sequestered fourth ventricle. Technical note." J NEUROSURG 80(4): 759-761.
59. Morina, D., et al. (2013). "Syringomyelia regression after shunting of a trapped fourth ventricle." Clin Pract 3(1).
60. Niwa, J., et al. (1992). "Cerebellar astrocytoma with repeated episodes of fourth ventricle isolation causing peritoneal shunt tube obstruction--case report." Neurol Med Chir (Tokyo) 32(6): 356-359.
61. Ogiwara, H. and N. Morota (2013). "Endoscopic transaqueductal or interventricular stent placement for the treatment of isolated fourth ventricle and pre-isolated fourth ventricle." CHILD'S NERV SYST 29(8): 1299-1303.
62. Oi, S., et al. (1999). "Neuroendoscopic surgery for specific forms of hydrocephalus." CHILD'S NERV SYST 15(1): 56-68.
63. Oi, S. and S. Matsumoto (1985). "Slit ventricles as a cause of isolated ventricles after shunting." Childs Nerv Syst 1(4): 189-193.
64. Oi, S. and S. Matsumoto (1986). "Pathophysiology of aqueductal obstruction in isolated IV ventricle after shunting." CHILD'S NERV SYST 2(6): 282-286.
65. Otsuki, T., et al. (1982). "Trapped fourth ventricle. Case report." ACTA NEUROCHIR 62(3-4): 259-264.
66. Owler, B. K., et al. (2001). "Lateral recess cysts in two cases of isolated fourth ventricle." CHILD'S NERV SYST 17(6): 363-365.
67. Pang, D., et al. (2005). "Progressive cranial nerve palsy following shunt placement in an isolated fourth ventricle. Case report." J NEUROSURG 102 PEDIATRICS(SUPPL. 3): 326-331.
68. Peraio, S., et al. (2018). "Endoscopic Management of Pediatric Complex Hydrocephalus." World Neurosurg 119: e482-e490.
69. Raouf, A. and I. Zidan (2013). "Suboccipital endoscopic management of the entrapped fourth ventricle: technical note." Acta Neurochir. 155(10): 1957-1963.
70. Rosenfeld, D. L., et al. (1995). "Transteutorial herniation of the fourth ventricle." PEDIATR RADIOL 25(6): 436-439.
71. Sagan, L. M., et al. (2006). "Endoscopic aqueductal stent placement for the treatment of a trapped fourth ventricle." J NEUROSURG 105 PEDIATRICS(SUPPL. 4): 275-280.
72. Salman, R., et al. (2022). "Radiologic and clinical outcome of isolated fourth ventricle following post-hemorrhagic hydrocephalus in children." CHILD'S NERV SYST 38(5): 977-984.
73. Sansone, J. M. and B. J. Iskandar (2005). "Endoscopic cerebral aqueductoplasty: A trans-fourth ventricle approach." J NEUROSURG 103 PEDIATRICS(SUPPL. 5): 388-392.
74. Say, I., et al. (2019). "Endoscopic Fourth Ventriculostomy: Suboccipital Transaqueductal Approach for Fenestration of Isolated Fourth Ventricle: Case Report and Technical Note." World Neurosurg 129: 440-444.
75. Schaumann, A., et al. (2021). "Neuroendoscopic surgery in neonates — indication and results over a 10-year practice." CHILD'S NERV SYST 37(11): 3541-3548.
76. Schulz, M., et al. (2013). "Endoscopic neurosurgery in preterm and term newborn infants - A feasibility report." CHILD'S NERV SYST 29(5): 771-779.
77. Schulz, M., et al. (2012). "Endoscopic treatment of isolated fourth ventricle: Clinical and radiological outcome." NEUROSURGERY 70(4): 847-858.
78. Scotti, G., et al. (1980). "The Isolated 4th Ventricle in Children - Ct and Clinical Review of 16 Cases." Am. J. Neuroradiol. 1(5): 419-424.
79. Sharma, R. R., et al. (2001). "CT stereotaxy guided lateral trans-cerebellar programmable fourth ventriculo-peritoneal shunting for symptomatic trapped fourth ventricle." Clin Neurol Neurosurg 103(3): 143-146.
80. Shin, M., et al. (2000). "Neuroendoscopic aqueductal stent placement procedure for isolated fourth ventricle after ventricular shunt placement. Case report." J NEUROSURG 92(6): 1036-1039.
81. Simonin, A., et al. (2015). "Cranial nerve palsies after shunting of an isolated fourth ventricle." BMJ Case Rep 2015.
82. Spennato, P., et al. (2007). "Neuroendoscopic treatment of multiloculated hydrocephalus in children." J. Neurosurg. 106(1): 29-35.
83. Spennato, P., et al. (2005). "Bilateral abducent and facial nerve palsies following fourth ventricle shunting: Two case reports." CHILD'S NERV SYST 21(4): 309-316.
84. Thakker, R. and A. Mohanty (2019). "Reversible Progressive Multiple Cranial Nerve Paresis in the Isolated Fourth Ventricle following Placement of Fourth Ventricle Shunt: Case Report and Review of the Literature." PEDIATR NEUROSURG 54(6): 405-410.
85. Tirado-Caballero, J., et al. (2021). "Cranial expansion and aqueductoplasty for combined isolated fourth ventricle and slit-ventricle syndrome: a surgical alternative." CHILD'S NERV SYST 37(3): 885-894.
86. Torres-Corzo, J., et al. (2004). "Trapped fourth ventricle treated with shunt placement in the fourth ventricle by direct visualization with flexible neuroendoscope." Minimally Invasive Neurosurg 47(2): 86-89.
87. Torrez-Corzo, J., et al. (2009). "Endoscopic management of brainstem injury due to ventriculoperitoneal shunt placement." CHILD'S NERV SYST 25(5): 627-630.
88. Tseng, J. S., et al. (2007). "Motor neuron disease-like syndrome secondary to trapped fourth ventricle and obstruction of cerebrospinal fluid pathway." Clin Neurol Neurosurg 109(4): 383-387.
89. Tyagi, G., et al. (2020). "Trapped fourth ventricle—treatment options and the role of open posterior fenestration in the surgical management." ACTA NEUROCHIR 162(10): 2441-2449.
90. Udayakumaran, S., et al. (2010). "Posterior fossa craniotomy for trapped fourth ventricle in shunted hydrocephalic children-long-term outcome." J Pediatr Neurosci 5(2): 174.
91. Udayakumaran, S., et al. (2009). "Unusual subacute diencephalic edema associated with a trapped fourth ventricle: Resolution following foramen magnum decompression." CHILD'S NERV SYST 25(11): 1517-1520.
92. Udayakumaran, S. and D. Panikar (2012). "Postulating the concept of compensated trapped fourth ventricle: A case-based demonstration with long-term clinicoradiological follow-up." CHILD'S NERV SYST 28(5): 661-664.
93. Upchurch, K., et al. (2007). "Endoscope-assisted placement of a multiperforated shunt catheter into the fourth ventricle via a frontal transventricular approach." Neurosurg Focus 22(4): E8.
94. Villavicencio, A. T., et al. (1998). "Avoiding complicated shunt systems by open fenestration of symptomatic fourth ventricular cysts associated with hydrocephalus." PEDIATR NEUROSURG 29(6): 314-319.
95. Zimmerman, R. A., et al. (1978). "Computed tomography of the trapped fourth ventricle." AM J ROENTGENOL 130(3): 503-506.
